# Household air pollution and cancers other than lung: a meta-analysis

**DOI:** 10.1186/s12940-015-0001-3

**Published:** 2015-03-15

**Authors:** Sowmya Josyula, Juan Lin, Xiaonan Xue, Nathaniel Rothman, Qing Lan, Thomas E Rohan, H Dean Hosgood

**Affiliations:** Department of Epidemiology & Population Health, Albert Einstein College of Medicine, 1309 Belfer Bronx, New York, USA; Division of Cancer Epidemiology and Genetics, National Cancer Institute, Bethesda, MD USA

**Keywords:** Solid fuels, Cervical cancer, Upper aero-digestive cancer, Meta-analysis, Risk factor

## Abstract

Household air pollution (HAP) from solid fuel combustion contributes to 2.6% of the global burden of disease. HAP emissions are an established lung carcinogen; however, associations with other cancer sites have not been fully explored. We conducted a meta-analysis of 18 case–control studies. Using fixed-effects models, utilizing the adjusted odds ratios (OR) and 95% confidence intervals (95% CI) from each study, we evaluated the association between HAP and cervical neoplasia (663 cases and 1747 controls) and upper aero-digestive tract cancers (6022 cases and 15 325 controls). We found that HAP was associated with cervical neoplasia (OR = 6.46; 95% CI = 3.12-13.36; 4 studies); oral (OR = 2.44; 95% CI = 1.87-3.19; 4 studies; 1000 cases/3450 controls); nasopharyngeal (OR = 1.80; 95% CI = 1.42-2.29; 6 studies; 2231 cases/2160 controls); pharyngeal (OR = 3.56; 95% CI = 2.22-5.70; 4 studies; 1036 cases/3746 controls); and laryngeal (OR = 2.35; 95% CI = 1.72- 3.21; 5 studies; 1416 cases/4514 controls) cancers. The elevated risk for esophageal cancer (OR = 1.92; 95% CI = 0.82-4.49; 2 studies; 339 cases/1455 controls) was non-significant. HAP was associated with cervical neoplasia among studies that accounted for HPV infection (OR = 9.60; 95% CI = 3.79-24.32) and smoking (OR = 4.72; 95% CI = 1.84-12.07). Similarly, our observed associations between HAP and upper aero-digestive tract cancers remained significantly elevated when analyses were restricted to studies that controlled for smoking. No significant publication bias was detected. Our results suggest that the carcinogenic effect of HAP observed for lung cancer may extend to other cancers, including those of the cervix and the upper aero-digestive tract. Further research is needed to confirm these associations in prospective studies.

**Electronic supplementary material:**

The online version of this article (doi:10.1186/s12940-015-0001-3) contains supplementary material, which is available to authorized users.

## Background

Around three billion people depend on solid fuels for household energy both in developed and developing countries [1,2]. Solid fuel consists of mainly coal and various forms of biomass, such as wood, crop residues and animal dung. The type of solid fuel used varies by geographic location, with coal being primarily used in China and biomass being primarily used in India and Africa. The annual global health burden from household air pollution (HAP) is approximately 3.9 million deaths [3], accounting for 2.6% of global burden of disease. In the year 2000 it was estimated that 16,000 premature deaths have been attributed to lung cancer alone [1]. Based on animal and human studies, the World Health Organization’s International Agency for Research on Cancer Working Group concluded that HAP from combustion of solid fuels, specifically coal, are carcinogenic to humans and particularly lung cancer [4-6]. Questions remain, however, as to if the carcinogenic potential of HAP extends beyond the lung.

Evidence suggests that polycyclic aromatic hydrocarbons (PAHs), a major HAP component, have carcinogenic properties on mucosal and endothelial lining of upper aero digestive tract from inhalation [7-9]. Studies have suggested that HAP may be associated with oral cancer and nasopharyngeal cancer [8-10]. Given that PAHs from smoking have been implicated in cervical cancer, the carcinogenic potential on the cervical and vaginal mucosal has been also explored for solid fuel use in human and animal models [11-13]. Given this background, we conducted a meta-analysis to assess the associations between HAP from all solid fuel types (coal, wood and mixed exposures) and upper aero-digestive cancer and cervical neoplasia as they have yet to be summarized in the literature.

## Methods

Studies that explored the association between upper aero- digestive and genital cancers with HAP, published between January 1970 and July 2014 were identified by searches of the PubMed and Science Citation Index databases using keywords related to indoor air pollution, cancer and cancer sites (Table 1). In addition, we manually searched the references from the articles that met our inclusion criteria.

**Table 1:** **Search strings and key words**

**Search Item**	**Keywords**
Indoor air pollution	‘IAP’ OR ‘indoor air’ OR ‘indoor environment’ OR ‘pollution’ OR ‘pollutant’ OR ‘exposure’ OR ‘fuel’ OR ‘fuels’ OR ‘coal’ OR ‘coals’ OR ‘charcoal’ OR ‘charcoals’ OR ‘cake’ OR ‘cakes’ OR ‘briquette’ OR ‘briquettes’ OR ‘solid fuel’ OR ‘solid fuels’ OR ‘biomass’ OR ‘anthracite’ OR ‘bituminous’ OR ‘fossil fuel’ OR ‘fossil fuels’ OR ‘lignite’ OR ‘subbituminous’ OR ‘stove’ OR ‘stoves’ OR ‘chula’ OR ‘chulla’ OR ‘oven’ OR ‘ovens’ OR ‘smoke’ OR ‘smoky’ OR ‘Wood’ OR ‘biomass’ OR ‘cooking oil’ ‘heat*’ OR ‘cook*’ OR ‘light*’ OR ‘burn*’ OR ‘fumes*’
Cancer site	head and neck’ OR ‘oral’ OR ‘oropharyngeal’ OR ‘pharynx’ OR ‘nasopharynx’ OR ‘hypopharynx’ OR ‘larngeal’ OR ‘esophageal’ OR ‘cervical’ OR ‘genital’
Cancer	‘cancer’ OR ‘cancers’ OR ‘carcinoma’ OR ‘carcinomata’ OR ‘neoplasm’ OR ‘neoplasms’ OR ‘tumor’ OR ‘tumors’ OR ‘tumours’ OR ‘tumour’

The inclusion criteria for our study were: (i) epidemiological studies (case–control and cohort studies) that examined the association between HAP and these cancers; (ii) the study population’s solid fuel use exposures were primarily derived from household cooking and/or heating and not from other forms of urban/outdoor air pollution or occupational exposures; and (iii) the results for the study population were not reported in another publication.

**Figure 1:**
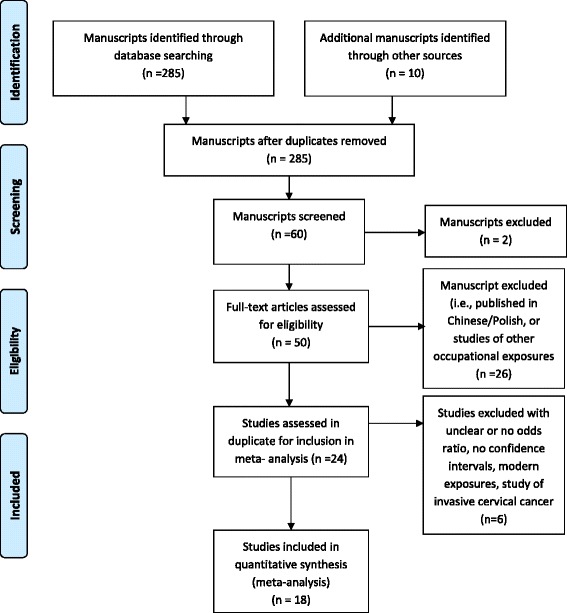
**Literature review and selection criteria for a meta-analysis on household air pollution and the risk of cancers other than the lung.**

Studies were excluded if they were (i) studies written in languages other than English and Spanish; (ii) animal studies; (iii) studies examining only the carcinogens implicated in HAP (i.e., benzo (a) pyrene); or (iv) focused on occupational exposure to solid fuel usage or exposure to cooking oil fumes. The initial keyword searches yielded 285 manuscripts of which 60 were selected for abstract review. Of these studies that met our inclusion criteria, 18 studies were reviewed. All of these studies were case–control studies by design, except for one nested case–control study. Refer to Figure 1 for further details.

Data related to study design, geographical location, population setting, case selection criteria, control selection criteria, exposure assessment methods, the number of cases and controls, participation rates, gender distribution, the type of fuel used, and risk of cancer associated with exposure, crude odds ratio (OR) and 95% confidence interval (95% CI), adjusted odds ratio (OR) and 95% CI, the variables adjusted for, and the limitations of the study were extracted from each study. Since there is no standardized method for HAP exposure assessment, we critically reviewed all the studies to determine the respective exposure assessment method. All studies utilized questionnaire-based methods to determine exposures either qualitatively or quantitative. ORs were extracted for all fuel types (i.e., wood, coal) provided by each study. For studies that provided multiple ORs based on various exposure groups, the OR representing the highest exposure group was selected. For example, the groups that experienced the longest duration of exposure or the groups with substantial fuel burning in poorly ventilated kitchens were selected. Since HAP attributed to coal exposures tend to have higher carcinogenic potential than HAP attributed to wood for lung cancer [6], we selected ORs related to coal exposure for our primary analyses when multiple ORs were provided. We also extracted coal and wood specific ORs for our exploratory analysis.

Table 2 summarizes the studies that were included in the meta-analysis. For quality assurance each study was reviewed twice by the first author and three of the manuscripts were randomly selected and extracted by the senior author to examine the robustness of the data extracted. There was 100% concordance between the two independent data extractions and between the extractions by the first and senior authors.

**Table 2:** **Summary of the 18 case–control studies included in a meta-analysis of the risk of cancers other than lung associated with household air pollution**

**Study**	**Setting**	**Cancer site studied**	**Cases**	**Controls**	**Type of case- control study**	**Definition of cases**	**Definition of controls**	**P.r. case**	**P.r control**	**% male case**	**% male control**
**Pintos (1998) [** 25 **]**	Brazil: 1987-89	Oral	373	1,568	Hospital based	Incident cancers. Excluded: salivary gland, nasopharynx cancers	Matched: sex, age, admission time. Excluded disease: cancers & mental	100	100	100	100
		Pharyngeal	217	1,568							
		Laryngeal	194	1,568							
**Sapkota (2012)** **[** 7 **]**	Europe: 2003	Oral	295	1,018	Hospital & population based	Age: 20–79, histologically confirmed	Matched: age, sex, residence, no cancer or tobacco disease	100	90 to 96	78	85
		Pharyngeal	201	1,040							
		Laryngeal	383	916							
		Esophageal	186	1,110							
**Sapkota (2008) ** **[** 14 **]**	India: 2001-04	Hypopharyngeal	513	718	Hospital & population based	Age < 80, residence > 1 year	Matched: age, sex, residence, no tobacco or alcohol diseases	100	##	87.5 ^	85
		Laryngeal	511	718							
**Dietz (1995)** **[** 24 **]**	Germany: 1989-92	Oral	100	400	Hospital based	Diagnosed cancer	Matched: sex, residence, age	##	##	##	##
		Pharyngeal	105	420							
		Laryngeal	164	656							
**Franco (1989)** **[** 23 **]**	Brazil: 1986-88	Oral	232	464	Hospital & population based	Malignancies of lip and salivary glands excluded.	Matched: sex, age, admission time. cancers, mental disorder excluded	100	100	87	87
**Maier (1997)** **[** 22 **]**	Germany: 1988-89	Laryngeal	164	656	Hospital based	Diagnosed with a time lapse < 3 years	Matched for age, residence	##	##	100	100
**Chang-Claude (1990)** **[** 21 **]**	China: 1988	Esophageal	153	345	Population based	Diagnosed cancer	No family history esophageal cancer or dysplasia	62	62	24	81
**Ferrera (2000)** **[** 20 **]**	Honduras: 1993-95	Invasive cervical	99	197	Hospital based	Age 20–65, no treatment, residence > 6 months. Poor mental health excluded	Matched: age, normal cervix, no history of hysterectomy or conization,	100	100	0	0
**Velema (2002)** **[** 18 **]**	Honduras: 1993-95	CIN	125	241	Hospital based	Diagnosis	Matched: age, normal cervix, Pap smear; never received treatment	100	100	0	0
**Wu (2004)** **[** 11 **]** ***	Taiwan: 1999-2000	CIN >2	116	197	Population based	Age 19 and above with CIN > 2	Matched: age, residence; negative pap smear	100	100	0	0
**Lee (2010)** **[** 12 **]**	Taiwan: 2003-08	CIN	324	1,200	Population based	All socioeconomic levels age 20 to 75, CIN > 1	Matched: residence, time of Pap smear, age; negative Pap smear	89	93	0	0
**Torres (2006)** **[** 13 **]**	Colombia: 2002-03	CIN	98	109	Hospital based	CIN 2, 3. Exclude: chemotherapy, chronic disease, family cancer, ill	Matched: age, residence; normal histology; no HPV treatment	##	##	0	0
**Feng (2009)** **[** 15 **]**	Africa: 2001- 04	NPC	636	615	Hospital based	Incident & prevalent cases, > 15 years, treated in public hospitals.	Matched: center, age, sex, urban/rural; non NPC	90	##	71	68
**Yu (1988)** **[** 26 **]**	China: 1984-86	NPC	128	174	Population based	Mothers of diagnosed NPC, age < 45	Matched: residence, race sex age	100	100	71	71
**Yu (1986)** **[** 8 **]**	Hong Kong: 1981	NPC	250	250	Population based	Incident cases, Chinese, age < 35	Matched: sex, age, ethnicity, marital status, education.	100	100	64	64
**Jeannel (1990)** **[** 16 **]**	Tunisia: 1986- 87	NPC	80	160	Population based	Diagnosed cases	Matched: sex, age, residence.	100	100	67	67
**Zheng (1994)** **[** 17 **]**	China: 1986	NPC	88	176	Population based	Incident NPC	Matched: sex, age, residence.	100	100	73	73
**Guo (2009)** **[** 19 **]**	China: 2004-05	NPC	1,049	785	Hospital based	Incident and prevalent cases	Matched: age, sex, residence; EBV antibodies +, no NPC; Excluded: minority, family members enrolled	##	##	72	72

## Not provided; CIN = Cervical Intraepithelial Neoplasm; NPC: Nasopharyngeal Carcinoma; p.r = participation rates; % males: gender distribution in the study; *** only nested case control study; setting = geographic area of study and year of participant enrollment; ^ average % for all cancers.

All statistical analyses were performed using the “meta” and “metafor” packages and in-house code developed in the R statistical language version 3.1.0. Heterogeneity among studies was determined using the Q test for heterogeneity. Summary ORs for cancers according to cancer site were then calculated using the adjusted OR and 95% CIs from each independent study. If the adjusted odds ratio was unavailable we used crude odds ratio. Using random and fixed-effects models, summary ORs were calculated for the overall effect of each cancer site and exposure of interest. Given that the numbers of studies were too few to obtain a stable estimate of variance of random effects, and that cancer site-specific ORs calculated using random-effects models were similar to those calculated using fixed-effects models, only results from fixed-effects models are presented. In addition, analyses were conducted to obtain summary ORs for studies that accounted for major known confounders, such as smoking status in upper aero-digestive cancers and HPV in cervical cancer. Exploratory analyses were also conducted based on specific type of fuel used. Publication bias was assessed by visual inspection of funnel plots (Additional file 1: Figure S1).

## Results

Eighteen studies met our inclusion criteria [7,8,11-26]. Table 2 presents a brief summary of these studies with the following information: Setting (country and year of study), type of cancer studied, case and control selection characteristics, criteria of inclusion and exclusion for cases and controls, participation rates for cases and controls, and percentage of males among cases and controls.

### Cervical neoplasia

**Figure 2:**
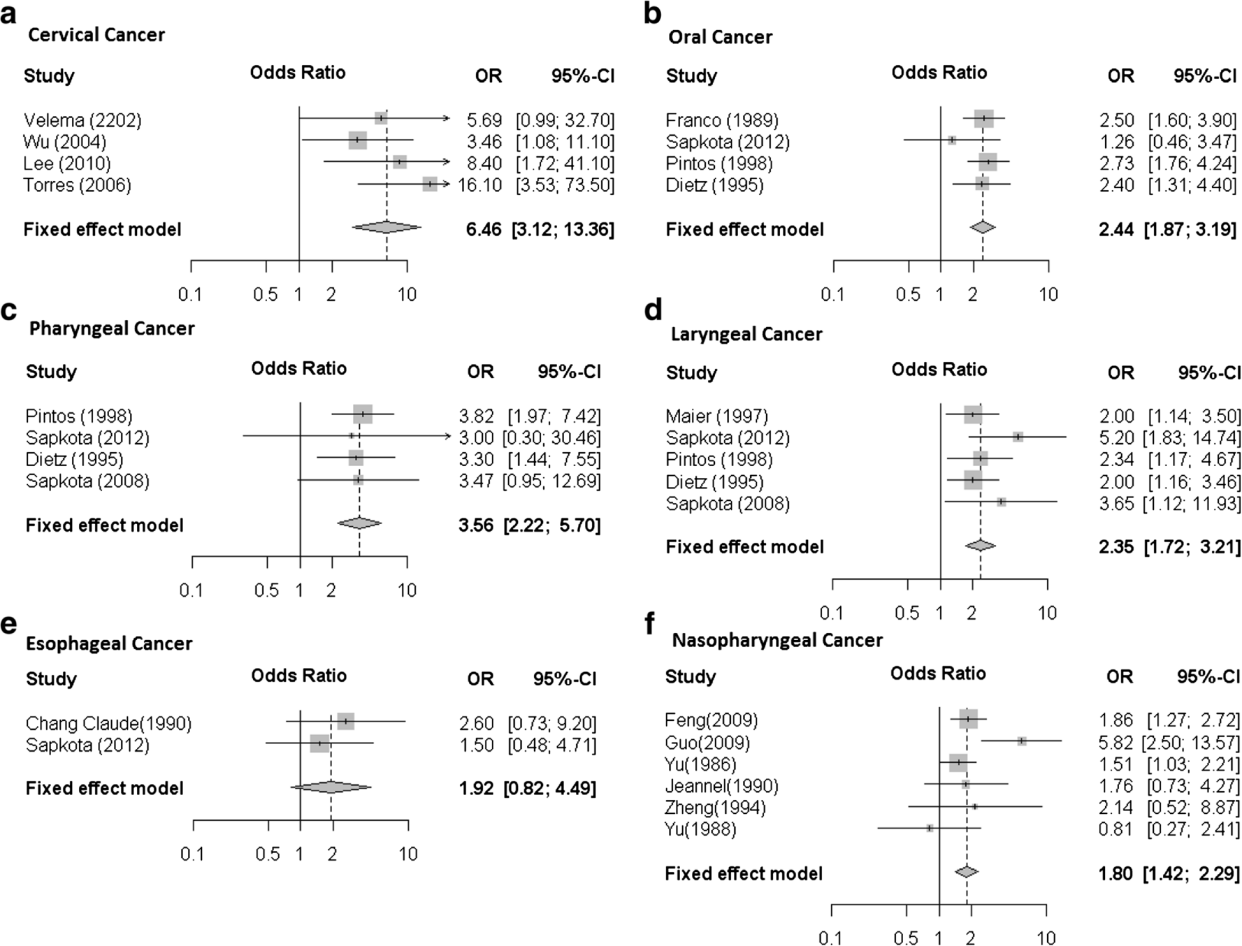
**Forest plots for the relative strength of the summary OR for for (a) Cervical cancer, (b) Oral cancer, (c) Pharngeal cancer (d) Laryngeal cancer (e) Esophageal cancer (f) nasopharyngeal cancer.**

Four studies evaluated the association between precursor lesions of cervical cancer and HAP (Table 3) [11-13,18]. Of the four studies on precursor lesions of cervical cancer, two were conducted in South America and two in Asia. The total number of cases of carcinoma in-situ (CIN) was 663, which was compared to 1747 controls. The summary OR for the 4 studies was 6.46 (95% CI =3.12- 13.36) (Figure 2a). There was no heterogeneity (p = 0.45) (Table 4).

**Table 3:** **Summary of studies analyzing cervical neoplasia risk associated with household air pollution**

**Study**	**Cases (N)**	**Controls (N)**	**Grade CIN**	**Exposure definition**	**Odds ratio**	**Account for HPV**	**Variables adjusted for**
**Velema (2002)** **[** 18 **]**	125	241	I, II, III	HPV +, CIN, wood smoke exposure 35+ years	5.69 (1.00 – 2.70)	Yes	HPV, education, parity, no: sex partners, age sexual debut
**Wu (2004)** **[** 11 **]**	116	197	>II	Age > 40, never used fume extractor	3.46 (1.08-11.10)	No	Age, education, smoking, no: prior pap smears, age at sexual debut, chefs
**Lee (2010)** **[** 12 **]**	324	1,200	I II, III	Cooked at age 20 to 40, HGSIL+, > 1 hour cooking, poor ventilation	8.40 (1.7 – 41.10)	Yes	Age, marital status, education, age at sexual debut, smoking, HPV DNA load and chef
**Torres (2006)** **[** 13 **]**	98	109	I, II, III	Exposure for > 45 years	16.10 (3.55 - 73.50)	Yes	HPV status, age at first sexual intercourse, yrs. education, rural vs urban

**Table 4:** **Meta-analysis results of literature evaluating the risk of cancers other than the lung associated with household air pollution**

	**Number of**			**Fixed effects model**		**Heterogeneity (p)**
**Cancer site**	**Studies**	**Cases**	**Controls**	OR*	**95% CI**	
**Cervical**	4	663	1747	6.46	3.12-13.36	0.45
**Oral**	4	1000	3450	2.44	1.87- 3.19	0.93
**Nasopharyngeal**	6	2231	2160	1.80	1.42- 2.29	0.06
**Pharyngeal**	4	1036	3746	3.56	2.22- 5.70	0.99
**Esophageal**	2	339	1455	1.92	0.82- 4.49	0.53
**Laryngeal**	5	1416	4514	2.35	1.72- 3.21	0.49

*Summary OR was calculated using fixed effects model for overall effect of each cancer site.

The two major risk factors for cervical neoplasia, HPV and smoking, have been independently controlled in some of the studies. The two studies that accounted for smoking remained significant (OR = 4.72; 95% CI = 1.84- 12.07) [11,12], as did the three studies that accounted for HPV status (OR = 9.60; 95% CI = 3.79- 24.32) [12,13,18]. Only one study accounted for both HPV and smoking status concurrently, and it observed an elevated risk for high grade lesions associated with cooking for more than an hour each day, with poor ventilation (OR =8.10; 95% CI = 1.70- 39.00), among women aged 20 to 40 years, who never smoked and the OR was adjusted for HPV DNA load [12].

### Upper aero-digestive cancer sites

Four studies (1000 cases/3450 controls) evaluated the association between oral cancer and HAP (Table 5), two were conducted in South America and two in Europe [7,23-25]. Summary OR was 2.44 (95% CI = 1.87-3.19) (Figure 2b) and there was no significant heterogeneity (p = 0.93) (Table 4). The summary OR for the three studies that accounted for smoking status, a significant risk factor for oral cancer, was found to be 2.56 (95% CI = 1.80-3.64) [7,24,25].

**Table 5:** **Summary of studies analyzing upper aero-digestive cancer risk associated with household air pollution**

**Cancer site**	**Study**	**Cases (N)**	**Controls (N)**	**Exposure definition**	**Odds ratio**	**Variables adjusted for**
**Oral**	Franco (1989) [23]	232	464	Exposure to wood stove	2.50 (1.60-3.90)	Age, sex, study site, admission period
	Sapkota (2012) [7]	295	1,018	Coal users for >50 years	1.26 (0.46-3.47)	Country, age, sex, BMI, pack years smoking, alcohol, and dairy, red meat, fruits, vegetables.
	Pintos (1998) [25]	373	1,568	Woodstoves for cooking and heating	2.73 (1.76- 4.24)	Socioeconomic, diet, employment, alcohol tobacco
	Dietz (1995) [24]	100	400	Single stove heating units with fossil fuel for > 40 years	2.40 (1.26 – 4.40)	Smoking and alcohol
**Nasopharyngeal**	Feng (2009) [15]	636	615	Kanoun (compact oven of charcoal) during childhood	1.86 (1.28-2.72)	Stratified: sex, center; adjusted: age, SES and diet
	Jeannel (1990) [16]	80	160	During childhood, kitchen in main room	1.76 (0.73 - 4.27)**	None
	Zheng (1994) [17]	88	176	Wood fire, poor ventilation	2.14 (0.94-8.87)**	None
	Yu (1988) [26]	128	174	Exposure to wood and dry grass	0.81 (0.27-2.41)**	None
	Yu (1986) [8]	250	250	Wood as cooking fuel	1.51 (1.03-2.21)**	None
	Guo (2009) [19]	1,049	785	>10 years exposure to wood fire	5.82 (2.50-13.57)	Solvent exposures, smoking
**Pharyngeal**	Pintos (1998) [25]	217	1,568	Woodstoves for cooking and heating	3.82 (1.96-7.42)	Socioeconomic, diet, employment place, alcohol and tobacco
	Sapkota (2012) [7]	201	1,040	Coal users for >50 years	3.00 (0.30- 30.46)	Country, age, sex, BMI, pack years, alcohol, and dairy, red meat, fruits, vegetables
	Dietz (1995) [24]	105	420	Single stove heating units with fossil fuel for >40 years	3.30 (1.43-7.55)	Smoking and alcohol
	Sapkota (2008) [14]	513	718	Coal use >50 years	3.47 (0.95-12.69)	Age, sex, center, SES, smoking, alcohol, chewing tobacco
**Esophageal**	Chang-Claude (1990) [21]	153	345	Wood fuel use before 1970	2.60 (0.07- 9.20)	Household status
	Sapkota (2012) [7]	186	1,110	Coal users >50 years	1.50 (0.48-4.71)	Country, age, sex, BMI, pack years, alcohol, and dairy, red meat, fruits, vegetables
**Laryngeal**	Maier (1997) [22]	164	656	> 40 years fossil fuel single stove	2.00 (1.10- 3.50)	Alcohol and tobacco
	Sapkota (2012) [7]	383	916	Coal users for >50 years	5.20 (1.84 -14.74)	Country, age, sex, BMI, pack years, alcohol, and dairy, red meat, fruits, vegetables
	Sapkota (2008) [14]	511	718	Coal use >50 years	3.65 (1.11-11.93)	Age, sex, center, SES, smoking, alcohol, chewing tobacco
	Pintos (1998) [25]	194	1,568	Woodstoves for cooking and heating	2.34 (1.17 - 4.67)	Socioeconomic, diet, employment place, alcohol and tobacco
	Dietz (1995) [24]	164	656	Single stove heating units with fossil fuel for > 40 years	2.00 (1.10 - 3.46)	Smoking and alcohol

**No adjusted OR provided in the study.

Six studies (2231 cases/2160 controls) estimated the risk of nasopharyngeal carcinoma from HAP. Four of these studies were conducted in Asia and two in Africa [8,15-17,19,26]. Summary OR was 1.80 (95% CI = 1.42- 2.29) (Figure 2f) and the test for heterogeneity was not significant (p = 0.06) (Table 4). Many of these studies explored the risk factors for nasopharyngeal cancer including dietary risk factors and ventilation in the kitchen. Only two of the studies provided an adjusted OR for the risk of HAP and nasopharyngeal cancer and the summary OR for these two studies remained statistically significant (OR = 2.25; 95% CI = 1.59- 3.18) [15,19]. The only study that accounted for EBV infection and smoking status observed an increased risk associated with HAP and nasopharyngeal cancer (OR = 5.82; 95% CI = 2.50 - 13.57) [19].

Four studies (1036 cases/3746 controls) assessed the risk of pharyngeal cancer associated with HAP (Table 5). These studies were conducted in Asia, South America and Europe [7,14,24,25]. The risk of pharyngeal cancer associated with HAP in these four studies was 3.56 (95% CI = 2.22- 5.70) (Figure 2c) and there was no substantial heterogeneity (p = 0.99) (Table 4). All 4 studies adjusted for smoking status.

Two studies (339 cases/1455 controls), which were conducted in Asia and Europe [7,21], evaluated esophageal cancer risk and HAP (Table 5). Summary OR for these was 1.92 (95% CI = 0.82- 4.49) (Figure 2e), with no heterogeneity (p = 0.53) (Table 4). Only one study adjusted for smoking and the adjusted OR was 1.50 (95% CI = 0.48- 4.71) [7].

Five studies (1416 cases / 4514 controls) evaluated the risk of laryngeal cancer associated with HAP (Table 5). Three of these studies were conducted in European countries, one in Asia and one in South America [7,14,22,24,25]. All the five studies accounted for smoking and the summary OR was 2.35 (95% CI = 1.72- 3.21) (Figure 2d), with no heterogeneity (p = 0.49) (Table 4).

### Type of fuel use

We conducted exploratory analyses to estimate fuel type-specific summary ORs (Table 6). These analyses are considered exploratory due to the limited number of studies included in the analyses.

**Table 6:** **Exploratory analyses based on type of fuel used**

	**Coal**		**Wood**	
**Cancer Site**	**Studies**	OR* (95% CI)	**Studies**	OR* (95% CI)
**Cervical**	1	3.46 (1.08- 11.10)	2	10.29 (3.27- 32.40)
**Oral**	1	1.47 (0.19- 11.67)	3	2.45 (1.81- 3.30)
**Nasopharyngeal**	1	1.86 (1.28- 2.72)	5	1.77 (1.07- 2.91)
**Pharyngeal**	2	3.35 (1.08-10.39)	3	2.56 (1.20- 5.49)
**Esophageal**	1	1.50 (0.48- 4.71)	2	2.13 (1.05 – 4.30)
**Laryngeal**	2	4.45 (2.03- 9.74)	3	1.54 (0.81- 2.94)

*Summary OR was calculated using fixed effects model for overall effect of each cancer site.

In precancerous cervical lesions, the summary OR for wood-specific exposure was 10.29 (95% CI = 3.27 – 32.40) [11,12]. For the upper aero-digestive cancers, the summary OR based on exposure to wood smoke depending on cancer site were as follows: oral cancer (OR = 2.45; 95% CI = 1.81- 3.30), pharyngeal cancer (OR = 2.56; 95% CI = 1.20- 5.49), laryngeal cancer (OR = 1.54; 95% CI = 0.81-2.94), esophageal cancer (OR = 2.13; 95% CI = 1.05- 4.30) and nasopharyngeal cancer (OR = 1.77; 95% CI = 1.07 to 2.91) (Table 6). Coal use was associated with increased risk of cervical, pharyngeal, and laryngeal cancers; however, these estimates were based on a limited number of studies.

## Discussion

Globally, 50% of all households and 90% of rural households continue to depend on solid fuels for cooking and heating [2]. An estimated 2.6% of the global burden of disease has been attributed to HAP [3]. With the existing evidence for the biological plausibility of HAP exposure potentially contributing to cervical neoplasia [27,28] and upper aero-digestive cancers [28,29] it is important to estimate this relationship.

Our meta-analysis of epidemiological studies confirms the results observed in animal studies [28,29], suggesting that the risk of upper aero-digestive cancers is associated with HAP. Our meta-analysis observed that HAP is associated with increased risk for oral, nasopharyngeal, pharyngeal, and laryngeal cancers. However, the increased risk noted with esophageal cancers was not statistically significant. The increased risks we observed seem to be independent of other risk factors such as smoking and age.

The carcinogenic potential of HAP has been previously evaluated. HAP is known to cause an increase household air levels of sulfur dioxide, carbon monoxide, fluorine, and known carcinogens such as polycyclic aromatic hydrocarbons (PAHs), benzene, arsenic, 1,3-butadiene and formaldehyde, [30,31]. These HAP constituents have been implicated in various malignant and nonmalignant diseases of organs on the route of exposure such as the respiratory tract, as well as the cardiovascular system [3]. Individual genetic variation and exposure to HAP is known to increase susceptibility to lung cancer [32].

The type of, and relative proportions of, carcinogens released varies by the type of fuel used [33]. Studies have found that combustion of coal increases the levels of PAHs, benzene, formaldehyde, silica and arsenic. The composition of these emissions vary from region to region [5]. Wood has been associated with elevated levels of PAHs, benzene, and 1,3-butadiene [5,33]. IARC concluded that HAP from combustion of coal was carcinogenic to humans (group 1) and that from wood was a probable carcinogen (group 2A) [4]. Studies found that coal smoke may potentially generate a higher carcinogenic potential than wood smoke for lung cancer [6]. However, research is needed to determine what constituent, or combination of constituents, of HAP is driving the carcinogenic potential. Given that the exposure depends on the type of fuel used [33] and the carcinogenic potential of the specific fossil fuel used [6], we explored the risk of our selected cancer sites by the type of fuel used. Although our analyses should be considered exploratory due to the limited number of fuel type specific results, it is interesting that our study suggestions that the risk associated with laryngeal cancers may be greater in populations with coal-specific exposures and the risk of cervical and oral cancers may be greater in those with wood-specific exposures.

Interesting evidence also emerges from our study that HAP is associated with increased risk of cervical neoplasia despite controlling for the risk factors such as HPV and smoking status [18]. There is limited mechanistic data available on how HAP may cause cervical neoplasia. However, in support of the hypothesis that HAP exposures may increase risk of cervical cancer, studies on cigarette smoking suggest that airborne nitrosoamines can be transmitted through blood to organs including cervix, thereby leading to cancer [29,34]. Therefore, it is conceivable that HAP exposures may mimic tobacco exposures with regard to cervical neoplasia risk. The causal mechanism for cervical neoplasia among HAP exposed warrants further investigation.

Our analysis has multiple strengths. This is the first meta-analysis to summarize the association between HAP and cancers other than lung. Though our study is a secondary data analysis we accounted for major risk factors to the extent possible by evaluating summary odds ratios based on study-specific ORs that adjusted for major confounders. Finally, the body of existing literature supports the biological plausibility for pathogenesis of these cancers. Even though we robustly reviewed the literature to identify all existing studies evaluating these associations, there were only a limited number of studies available for analysis. Another potential limitation is the file drawer effect of unpublished studies; however, our results are unlikely to be influenced by the file drawer effect, especially for upper aero-digestive tract, given the recent interest in HAP in relation to lung cancer [4]. We note, however, that even though the visual inspection of the funnel plots did not suggest the presence of publication bias, we cannot definitively rule out the possibility of publication bias. There were fewer than the 10 studies for each cancer site in this meta-analysis, which is the number of studies needed to draw formal conclusions on publication bias using the Begg’s test [35].

## Conclusions

Our results support the hypothesis that HAP is associated with the risk of cancers other than lung cancer. Our meta-analysis, however, should be considered hypothesis-generating until these associations are evaluated prospectively in order to overcome the limitations of the case–control design that was employed by virtually all of the studies included here.

## Additional file

Additional file 1: Figure S1. Funnel plots for cancer site specific analyses.

## Abbreviation

95% CI: 95% confidence intervals
OR: Adjusted odds ratios
CIN: Carcinoma in-situ
OR: Crude odds ratio
HAP: Household air pollution
HPV: Human papilloma virus
PAHs: Polycyclic aromatic hydrocarbons
