# Supplementary material for: Household air pollution and cancers other than lung: a meta-analysis
Source: Environ Health. 2015 Mar 15;14:24. doi: 10.1186/s12940-015-0001-3 (PMC4377187; doi:10.1186/s12940-015-0001-3)

**Supplemental Figure 1:** **Funnel plots for cancer site specific analyses**


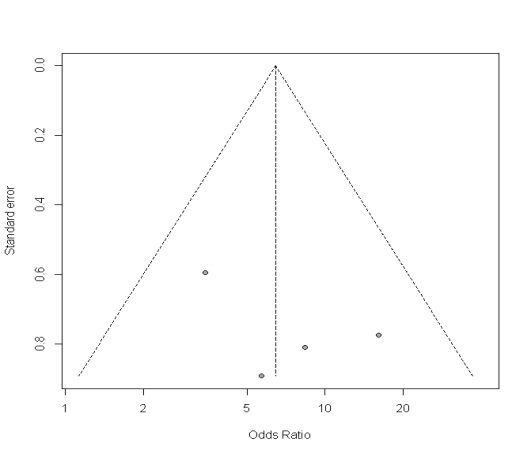

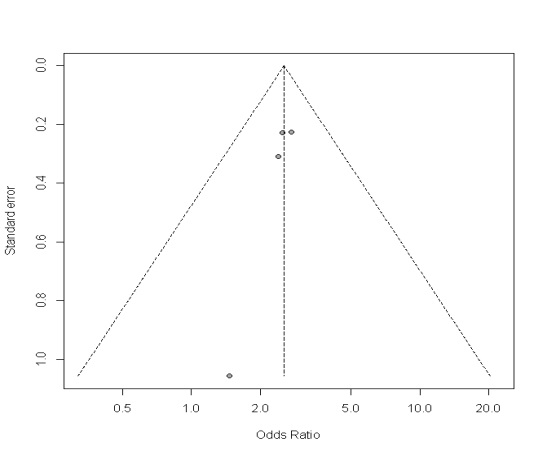


Laryngeal Cancer Fig 3d

Pharyngeal Cancer Fig 3c

Oral Cancer Fig 3b

Cervical Cancer Fig 3a


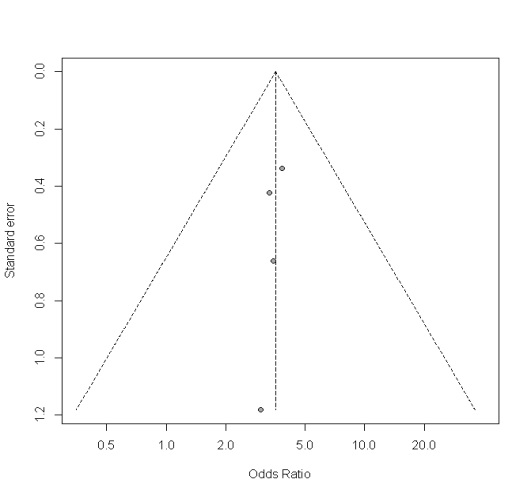

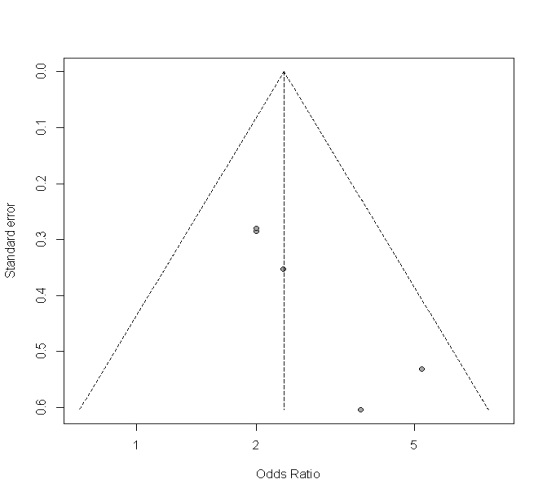


Nasopharyngeal cancer Fig 3f

Esophageal Cancer Fig 3e


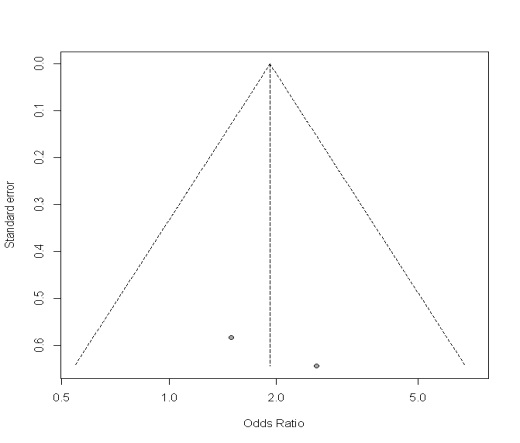

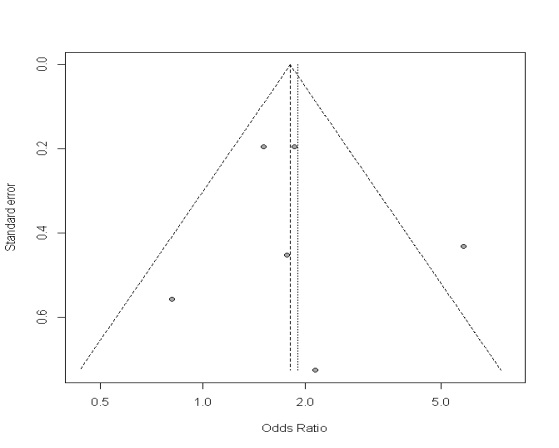

Supplement: Additional file 1: Figure S1. — Funnel plots for cancer site specific analyses. [file 12940_2015_1_MOESM1_ESM.docx]
